# A Digital Intervention to Improve Mental Health and Interpersonal Resilience in Young People Who Have Experienced Technology-Assisted Sexual Abuse: Protocol for a Nonrandomized Feasibility Clinical Trial and Nested Qualitative Study

**DOI:** 10.2196/40539

**Published:** 2023-03-21

**Authors:** Sandra Bucci, Filippo Varese, Ethel Quayle, Kim Cartwright, Matthew Machin, Pauline Whelan, Prathiba Chitsabesan, Cathy Richards, Victoria Green, John Norrie, Matthias Schwannauer

**Affiliations:** 1 Division of Psychology and Mental Health School of Health Sciences, Faculty of Biology, Medicine and Health, Manchester Academic Health Science The University of Manchester Manchester United Kingdom; 2 Greater Manchester Mental Health NHS Foundation Trust Manchester United Kingdom; 3 School of Health in Social Science The University of Edinburgh Edinburgh United Kingdom; 4 Division of Informatics, Imaging & Data Sciences School of Health Sciences, Faculty of Biology, Medicine and Health, Manchester Academic Health Science The University of Manchester Manchester United Kingdom; 5 Pennine Care NHS Foundation Trust Greater Manchester United Kingdom; 6 University College London London United Kingdom; 7 NHS Lothian Edinburgh United Kingdom; 8 Marie Collins Foundation Manchester United Kingdom; 9 Population Health Sciences Usher Institute, Centre for Population Health Sciences Edinburgh Clinical Trials Unit Edinburgh United Kingdom

**Keywords:** technology-assisted sexual abuse, digital, young people, abuse, eHealth, mobile health, mHealth, mobile phone

## Abstract

**Background:**

No evidence-based support has been offered to young people (YP) who have experienced technology-assisted sexual abuse (TASA). Interventions aimed at improving mentalization (the ability to understand the mental states of oneself and others) are increasingly being applied to treat YP with various clinical issues. Digital technology use among YP is now common. A digital intervention aimed at improving mentalization in YP who have experienced TASA may reduce the risk of revictimization and future harm and make YP more resilient and able to manage distress that might result from TASA experiences.

**Objective:**

In this paper, we describe a protocol for determining the feasibility of the i-Minds trial and the acceptability, safety, and usability of the digital intervention (the *i-Minds* app) and explore how to best integrate i-Minds into existing routine care pathways.

**Methods:**

This is a mixed methods nonrandomized study aimed to determine the feasibility, acceptability, safety, and usability of the intervention. Participants aged between 12 and 18 years who report distress associated with TASA exposure will be recruited from the United Kingdom from the National Health Service (NHS) Trust Child and Adolescent Mental Health Services, sexual assault referral centers, and a web-based e-therapy provider. All participants will receive the i-Minds app for 6 weeks. Coproduced with YP and a range of stakeholders, the i-Minds app focuses on 4 main topics: mentalization, TASA and its impact, emotional and mental health, and trauma. A daily prompt will encourage YP to use the app, which is designed to be used in a stand-alone manner alongside routine care. We will follow participants up after the intervention and conduct interviews with stakeholders to explore the acceptability of the app and trial procedures and identify areas for improvement. Informed by the normalization process theory, we will examine barriers and enablers relevant to the future integration of the intervention into existing care pathways, including traditional clinic-based NHS and NHS e-therapy providers.

**Results:**

This study was approved by the Research Ethics Board of Scotland. We expect data to be collected from up to 60 YP. We expect to conduct approximately 20 qualitative interviews with participants and 20 health care professionals who referred YP to the study. The results of this study have been submitted for publication.

**Conclusions:**

This study will provide preliminary evidence on the feasibility of recruiting YP to a trial of this nature and on the acceptability, safety, and usability of the i-Minds app, including how to best integrate it into existing routine care. The findings will inform the decision to proceed with a powered efficacy trial.

**Trial Registration:**

International Standard Randomised Controlled Trial Number Registry (ISRCTN) ISRCTN43130832; https://www.isrctn.com/ISRCTN43130832

**International Registered Report Identifier (IRRID):**

DERR1-10.2196/40539

## Introduction

### Background

Child sexual abuse (CSA) is a major risk factor for many health risk behaviors as well as physical and mental health problems. CSA negatively impacts developmental trajectories, educational attainment, occupational prospects, and communities more broadly, at huge societal and economic costs [1,2]. For young people (YP), using the internet is a routine part of daily life, but it can place them at risk for various forms of CSA. Technology-assisted sexual abuse (TASA) can occur through any device connected to the internet and across multiple platforms and apps. YP can be coerced into sharing sexual images of themselves, taking part in sexual activities via a webcam or smartphone, or having sexual conversations by text (“sexting”). Web-based grooming, abuse, and exploitation may also lead to contact abuse; contact abuse cases increasingly involve web-based elements (eg, the production and distribution of images). Offline and web-based sexual abuse is not mutually exclusive in terms of risk behaviors or harm, but there is evidence of additional risks of TASA afforded by the internet and unique social and psychological harms associated with TASA [3,4].

COVID-19 has led child protection organizations to urge governments, tech companies, educators, and parents to be alert, take urgent measures to mitigate potential risks, and ensure that YP engage in web-based interactions in a safe and positive way. In the United Kingdom, an Online Safety Bill has been published and further consultation will be brought into law in 2022. The bill acknowledged the need for new legislation to make the internet a safer place for everyone, especially children, while protecting their right to freedom of expression on the web. The sharp increase in TASA, even before COVID-19, had already caused increased pressure on health care services, education, and government systems. Recent data show an increase of 34% in the demand for mental health support from YP during the pandemic [5]. The distinctive nature of TASA relative to other forms of abuse is recognized in the current National Institute for Clinical Excellence (NICE) guidelines for responding to child abuse and neglect [1]. The NICE found no evidence-based interventions for improving the mental health and well-being of YP who have experienced TASA (YP-TASA) and recommended further research on the efficacy of interventions aimed at improving well-being and relationships and preventing further harm following internet-facilitated abuse. The efficacy of interventions that could improve well-being and prevent further harm in YP-TASA remains an unmet research need.

### Theoretical Framework

Multiple factors are likely involved in vulnerability to being exposed to TASA. A relevant risk factor is a young person’s ability to accurately estimate others’ intentions and motivations when engaging in a web-based environment. This ability to understand what is going on in our own minds as well as in other people’s minds (in terms of thoughts, intentions, desires, and beliefs) is known as mentalization [6]. Mentalization is the ability to attend to and reflect on the mental states in ourselves and in others and consequently understand our own actions and those of others on the basis of intentional mental states. An inverse relationship exists between emotional arousal and mentalization [7]. YP who are distressed or have difficulties with regulation because of being victimized, abused, or exploited on the web may be at the greatest risk of developing difficulties in mentalizing, increasing the likelihood of repeated victimization and harm [8]. Furthermore, social anxiety (often experienced by YP who are groomed on the web) has been found to be associated with mentalization difficulties [9]. People’s assumptions about the intentions and motives of others are usually based on the verbal and nonverbal cues of real-life interactions. When we communicate in a web-based environment, signals of empathy and understanding are transmitted more opaquely [10-12], and mentalizing, which is already compromised in vulnerable YP, can be even further affected. Difficulty mentalizing can compromise the evaluation of risk and assumed trust in web-based communications [13] and might therefore represent a valuable target for interventions aimed at reducing risk in YP who have already been exposed to TASA. Indeed, difficulties in mentalizing processes have been linked to greater vulnerability to a range of mental health problems that are common among TASA survivors, including depression, anxiety, eating disorders, and shame [14,15].

A recent systematic review [14] has highlighted mentalization-based therapy (MBT), a therapeutic approach that specifically aims to improve mentalizing capacity and consequently affect regulation and psychological distress, as a promising treatment approach across a wide range of clinical presentations, including groups that have previously shown a limited response to psychological therapy (eg, adolescents who self-harm). The efficacy of MBT has been more extensively trialed in adult mental health; however, a growing evidence base is emerging for the efficacy of MBT in YP. For example, in a randomized controlled trial with adolescents who self-harmed, Rossouw and Fonagy [16] found that MBT for adolescents was more effective than treatment as usual, with a recovery rate of 44% in comparison with 17%. A pilot randomized controlled trial conducted by Griffiths et al [17] found that a brief (12 weeks) mentalization-based intervention was acceptable in YP enrolled in Child and Adolescent Mental Health Services (CAMHS) and was associated with significant treatment effects across a range of outcomes, including anxiety, self-harm behaviors, and the ability to regulate distressing emotions. Therefore, improved mentalization capacity following a mentalization-based psychological intervention might result in improvements in two key intervention targets for YP-TASA: (1) reducing the risk of revictimization and future harm and (2) improving the emotional and mental well-being of users who might experience current distress due to TASA experiences.

### The Role of Digital Interventions

Although psychological interventions for YP can be delivered across multiple modalities, digitally mediated interventions represent an acceptable way to support YP and overcome some central limitations of traditional clinic-based services. Recent systematic reviews and meta-analyses have demonstrated that digitally mediated psychological interventions represent effective treatment options for improving the mental health and well-being of YP across a range of problems [18], including computerized cognitive behavior therapy targeting depression and anxiety, which have been most commonly examined. Existing feasibility, acceptability, and efficacy studies of digital interventions indicate that they are acceptable across genders [19], impact behavior as well as mood [20], and are safe for vulnerable YP [21]. Long waiting times in CAMHS indicate that there are significant delays in help being offered, preventing timely access to support, potential exacerbation of problems brought about by TASA exposure, and increased risk for repeated victimization in the interim. When effectively integrated within existing care pathways for YP, a digital intervention for YP-TASA has the potential to (1) scale-up access to support and tackle the overwhelming demand for services to provide timely therapeutic input and (2) ensure support can be implemented “in the flow of daily life” and in a format that is both familiar to YP and unconstrained by location and time, allowing access to therapeutic strategies even when traditional means of support are not available.

### This Study

The aim of our National Institute for Health and Care Research (NIHR)–funded i-Minds clinical feasibility trial was to assess the feasibility, acceptability, safety, and usability of a theory-driven, coproduced, mentalization-based digital health intervention (the “i-Minds” app) for YP-TASA. This trial represents the third phase in the development and testing of the i-Minds app. In phase 1, we interviewed health care professionals (HCPs) across both Northwest England and Edinburgh, Scotland, to inform them of the content of the i-Minds app and the protocol design of the trial. We explored the following issues: (1) HCPs experiences of adapting face-to-face interventions to a web-based environment, (2) their understanding of TASA and current service approaches to supporting YP-TASA, (3) specific issues HCPs face when working with YP-TASA, (4) previous experience of mentalization approaches in clinical work, (5) advantages and disadvantages of delivering psychological approaches using digital tools, (6) perceived barriers or facilitators to implement digital tools in clinical practice, and (7) uptake of a digital health intervention of this nature with this client group and our proposed trial procedures (results currently being prepared for publication). We also held monthly to bimonthly participatory workshops with a range of stakeholders, including those with lived experience, YP, parents and professionals, and nationally representative stakeholders, to inform the design and functionality of the digital health intervention and our proposed trial procedures. In phase 2, the University of Manchester Digital Health Software (DHS) team built the i-Minds app over an 8-month period of agile development and testing alongside qualitative and participatory design work. In the third phase, using a nonrandomized open trial design, we will test whether the i-Minds app is feasible, acceptable, and safe for YP-TASA.

Regarding consent, where prospective participants expressed an interest in taking part in the study, their clinician obtained the individual’s written or verbal consent to pass on their name and contact details to the research team. Once it has been established that a prospective participant is interested in taking part in the study and has given permission to be contacted by the research team, the researcher will contact the prospective participant to explain the study to them in detail and give them the opportunity to ask questions. The researcher will explain that participation is voluntary and that they can withdraw consent at any point during the study without giving a reason, which will not impact them continuing to access standard care within the referring service (as the i-Minds app is designed to be used in addition to, not in place of, standard care) or other sources of support they might access simultaneously.

Personally identifiable data are collected by the local recruitment site, separate from the research database. The research data were deidentified.

YP-TASA participants will be remunerated £40 (US $50) for their time to complete the baseline assessment (£20 [US $25]) and the follow-up (postintervention) assessment (£20 [US $25]). Participants who also participated in the nested qualitative interview study will receive a further £20 (US $25) for their time. This amount was based on NIHR INVOLVE guidelines. YP who do not have access to a smartphone will be provided with one, and their data network charges will be covered for the 6-week intervention period. HCPs who participated in the nested qualitative interview study will not receive payment for their participation.

### Objectives

The primary study objectives were to determine (1) whether it is feasible to deliver the i-Minds app for YP-TASA, including recruitment and retention to the trial, and identifying primary and secondary outcome measures; (2) whether the i-Minds app is acceptable to users; (3) whether the i-Minds app is usable and safe; and (4) how best to integrate the app into existing National Health Service (NHS) and web-based mental health and sexual abuse service care pathways.

## Methods

### Study Setting

The trial is funded by the NIHR Health and Social Care Delivery Research program. The study took place across the CAMHS and sexual assault referral centers in Greater Manchester (England) and Edinburgh (Scotland). We also aimed to recruit participants from an NHS-commissioned national (England) e-therapy provider (Kooth).

### Ethics Approval

The study was approved by the National Research Ethics Committee (REC) of West Scotland REC 4 (approval number 22/WS/0083).

### Study Design

#### Overview

This was a multicenter mixed methods study comprising two components: (1) a nonrandomized feasibility clinical trial with the aim of recruiting a target sample of 60 YP-TASA across recruitment sites. This sample size was shown to be sufficient to pilot test the feasibility and acceptability of an intervention of this nature. All participants will have access to the app and will be included in all analyses for the feasibility clinical trial. To evaluate who might benefit from using the i-Minds app, we will recruit participants with varied characteristics wherever possible (eg, ethnicity, sexual orientation, gender, TASA experiences); (2) a combination of quantitative data on adoption, uptake and use (aligned with the AMUsED framework [22]), and qualitative data from semistructured interviews guided by the Normalization Process Theory (NPT) with participants (n=approximately 20) and HCPs who referred YP-TASA to the trial (n=approximately 20) and service managers from the referring service provider (n=approximately 10) who referred to the trial will explore the acceptability and usability of the i-Minds app.

#### Eligibility

The participant inclusion and exclusion criteria are presented in Textboxes 1 and 2, respectively.

**Inclusion criteria.**

- Young people (YP) who have experienced technology-assisted sexual abuse (TASA) participants
  - Aged 12 to 18 years
  - Have been exposed to (TASA) and report associated distress
  - Are receiving support from National Health Service Child and Adolescent Mental Health Services, sexual assault referral centers or e-therapy provider (Kooth) and will continue to be actively supported by the provider over the duration of the trial
  - Willing to use an app designed to support YP who have experienced TASA
  - Proficient in speaking and writing in English
  - Capacity to consent
  - Consent to providing their username to the research team (Kooth participants only)
- Health care providers or service manager participants (qualitative interviews)
  - The member of the young person’s direct care team who referred the young person to the feasibility clinical trial
  - The manager at the service that referred the YP to the feasibility clinical trial
  - Able to understand and speak English

**Exclusion criteria.**

- Young people who have experienced technology-assisted sexual abuse people participants
  - Insufficient verbal and written command of English
  - Moderate learning difficulties (as assessed by their direct care team)
  - Are at risk of current or recent suicidality
- Health care professional participants
  - None (other than not meeting the inclusion criteria)

#### Recruitment

##### Feasibility Clinical Trial

The research team will liaise with the CAMHS and sexual assault referral centers teams and the e-therapy provider Kooth to provide information about the study via presentations at team meetings and study information leaflets, referral and consent to contact forms, and copies of the relevant participant information sheet. Clinical staff will be encouraged to identify potentially eligible participants from their caseload. Participants may also be identified by placing poster advertisements in waiting rooms or via digital spaces (via the digital magazine used by the e-therapy provider Kooth). The advertisement will invite interested prospective participants to ask for information about the study from a member of their clinical team.

After obtaining informed consent, potential participants will be provided with a participant information sheet for reading. Prospective participants aged 12-15 years recruited from the English NHS recruitment site will be provided with a bespoke participant information sheet to give to at least 1 of their parents or caregivers. The parent or caregiver will have the ability to opt their child out of taking part in the study (by contacting the research team or the child’s clinical team or by completing an opt-out form), should they not wish their child to take part. Prospective participants aged 12-15 years recruited from the Scottish site will be asked if they would like their parents to be given a participant information sheet.

##### Nested Qualitative Study: YP-TASA Process

Participants who participated in the feasibility clinical trial and who consented to participate in the qualitative study will be eligible to participate in qualitative interviews, which aim to understand how participants experienced the i-Minds app and its acceptability. Participants will be invited to the qualitative interview study when information is provided, and consent will be obtained for the feasibility clinical trial. Participants will be approached directly by the research team to participate in the interview upon discontinuation from accessing and using the app or completion of using it. Therefore, participants who do and do not complete the feasibility clinical trial in its entirety will be asked to participate in the interview. Participants will be selected according to a sampling framework to capture varied demographics, experiences of TASA, and levels of engagement with the i-Minds app.

##### Nested Qualitative Study: HCPs or Service Managers

As trial participants start completing postintervention assessments, a research worker will contact and invite a target sample of approximately 20 HCPs who referred YP-TASA to the feasibility clinical trial and 10 service managers from the referring service provider to participate in a semistructured interview (postintervention). A list of referring clinicians or managers will be kept separate from the research data.

### Consent

Trained members of the research team will receive consent. In both the quantitative feasibility clinical trial and nested qualitative interview study, fully informed consent will be sought from YP and HCPs or service managers and obtained before data collection and when participants access and use the app. Health Research Authority (HRA)– or REC-approved copies of age-appropriate participant information sheets and consent forms will be used for prospective YP, including those aged 12-15 years and 16-18 years. Prospective participants aged 12-15 years who are recruited in England will be provided with a participant information sheet to give to at least 1 of their parents or caregivers, who will be given the opportunity to opt for their child to not wish their child to take part in the study. Parents or caregivers of 12-15 year olds recruited from our Scottish site will be given a copy of the participant information sheet if the young person wishes. Parents or caregivers of prospective participants aged 12-15 years in Scotland will not be able to opt out of the study if the prospective participant is Gillick competent.

YP are provided with information about both the feasibility clinical trial and the qualitative study in one age-appropriate participant information sheet given to them before taking part in the feasibility clinical trial. They were asked to consent to participate in the qualitative study as part of the consent process for the feasibility clinical trial, but continued consent was checked if they were invited to participate in the interview.

The researcher will explain to all potential participants that participation is voluntary and that they can withdraw their consent at any point during the study without providing a reason. It will be made clear to YP that, as the intervention is not in place of standard care (it is in addition to standard care), withdrawal from the intervention will not impact their ability to continue to access standard care within the referring service or other sources of support they might access contemporaneously. Prospective participants in both the feasibility clinical trial and qualitative study will be given multiple options for documenting their informed consent, including (1) a signed hard copy of the consent form by standard mail, (2) returning a digitally signed copy of the consent form by email (encrypted), and (3) recording their consent on an encrypted audio file that will be stored separately from any research data collected from them. The participant’s general practitioner or care team will be provided with a letter to inform them that a young person is taking part in the study.

Participants will be given at least 48 hours to decide whether to participate. If a participant who has provided informed consent loses the capacity to consent during the study, the participant will be withdrawn from the study. Identifiable data already collected with consent will be retained and used in the study. No further data will be collected or any other research procedures will be carried out on or in relation to the participant. The withdrawn participants will be referred to their service provider so that appropriate follow-up care and action can take place.

### Data Collection and Management

Quantitative and qualitative data were collected through psychometric measures and interviews. The initial interview will involve the completion of self-reported sociodemographic questions, technology use, and clinical or service details. A range of validated self-report scales will measure key intervention targets and outcomes of the intervention both before (week 1) and after using the app (weeks 7-9 post baseline), including reflective function (mentalization), problematic internet use, emotional distress, internet abuse-related distress, emotion regulation, interpersonal sensitivity, attitudes and views toward close interpersonal relationships, and resilience. Multimedia Appendix 1 describes the schedule of the assessments at the 2 assessment time points.

Measures will be completed via the web-based Qualtrics survey system, capturing all the measures in the case record form, or, where this is not possible, a paper or hard-copy version of the case record form will be used. Demographics, clinical, and web-based abuse-related distress measures will be completed with support from the research worker (participants will be given the choice to complete the remaining self-report measures either on their own or with continued support from the researcher). The assessments of reflective function and emotion regulation will be administered next, with the remaining measures completed in a randomized order. All data (ie, demographic data, clinical data, self-report questionnaires, data related to trial administration, and procedures) will be entered and managed on the REDCap (Research Electronic Data Capture; Vanderbilt University) platform [23], a secure clinical trial management system. Data will be stripped of any identifying information and each participant will be assigned a unique participant ID. All information obtained is confidential. Research workers at each participating site will input data collected via Qualtrics (Qualtrics International Inc) into the REDCap database. In the case of paper or hard copy–completed questionnaires, research assistants will (1) scan paper copies for digital preservation of all research completed as part of the study and (2) enter questionnaire scores on the REDCap database.

Qualitative interviews will be audio-recorded for transcription and analytic purposes, using recording devices that enable encryption at the point of data collection. All interviews were anonymized at the time of transcription, and all identifying details were removed. Audio-recorded consent (including participants’ names) will be recorded on a separate audio file so that this information cannot be directly linked with the interview transcripts or audio recordings.

### Outcomes

#### Primary Outcome

The primary objective of the trial was to determine the feasibility and acceptability of delivering the i-Minds app for YP-TASA. No formal attempt has been made to test the effectiveness of the intervention which will be conducted.

##### Feasibility

We will collect detailed recruitment and retention data congruent with all relevant fields of the CONSORT (Consolidated Standards of Reporting Trials) statement for feasibility studies [24], including (1) the number of eligible participants consenting; (2) the total number recruited, including information about recruitment setting to inform the sampling strategy of a future definitive trial; (3) completeness of outcome measures; (4) the number lost to follow-up; and (5) number of services offered the intervention. To evaluate the extent to which YP-TASA engaged with i-Minds, we collected data on the (1) proportion of participants completing the intervention, (2) dropout rates and reason for withdrawal, and (3) platform use and engagement data using Matomo click analytics software [25] with use analysis guided by the AMUsED framework [22].

##### Acceptability

To understand how the intervention is experienced and how acceptable the app and trial procedures are, we will conduct in-depth qualitative interviews with a proportion of YP-TASA who will be selected according to a sampling framework to capture varied demographics, experiences of TASA, and levels of engagement with the i-Minds app. Interviews will be conducted in rounds of approximately 5 participants to allow for iterative analysis and inform further sampling. Topic guides developed with input from our advisory groups will be used to examine (1) whether i-Minds met expectations; (2) what level of support is needed to facilitate engagement with i-Minds; (3) overall impressions of i-Minds (enjoyable, usability, and satisfaction); (4) what participants liked and did not like about the intervention in terms of content and usability of the platform; (5) how it helped and did not help; (6) what changes they would make; (7) barriers to participation or engagement, including the perceived burden of the research assessment procedures; and (8) acceptability of trial procedures.

To examine barriers and enablers (and unintended consequences) to integration and uptake of the intervention into existing care pathways, we conducted qualitative interviews with a target sample of 20 HCPS who referred to the trial and 10 service managers to examine specific questions around (1) ways in which we can maximize uptake, utility, user experience, acceptability, satisfaction, and reach of the platform; (2) how the platform can be locally adapted and translated into practice; (3) HCPs’ views on referral routes to a digital intervention within existing care pathways; and (4) strategic perceptions about the relative advantage of the digital platform and its wider transactability (whether it can be scaled up). Before taking part in the interview, HCPs will be asked to complete some brief demographic questions (eg, age, profession, and years of professional experience).

Topic guides were developed in collaboration with our lived experience, parents or caregivers, and professional advisory groups. We anticipate all qualitative interviews to take approximately 1 hour, depending on the extent to which the participant requires breaks or further support or guidance and includes full debriefing.

#### Secondary Outcomes

The secondary objectives of this trial were to explore whether the i-Minds app brings about clinically meaningful changes in outcomes (described in Multimedia Appendix 1), differences in engagement and attrition, and potential clinical benefits across key demographic groups (eg, lesbian, gay, bisexual, transgender, queer, and others [LGBTQ+] and Black, Asian, and minority ethnic [BAME]), demographics and service differences across recruitment sites (Manchester, Edinburgh, or Kooth), and barriers and enablers to integration and uptake into existing mental health care provider pathways. We will also explore the safety and usability of the app and our trial procedures.

##### Safety

We will collect detailed adverse event reports using standardized operating procedures in line with our study sponsor and HRA safety reporting procedures.

Any adverse event observed over the course of the research will be documented and reported according to a bespoke standard operating procedure that will fully comply with appropriate HRA safety reporting procedures, sponsor requirements, and local research and development policies of participating NHS organizations. All adverse events will be monitored by an independent Data Monitoring and Ethics Committee (DMEC) that aims to meet on 3 occasions (and more frequently if needed) over the course of the trial period. An adverse event log file will be created to systematically record occurrences with reference to an a priori defined list of anticipated and unanticipated adverse events.

##### Usability

As this was a feasibility clinical trial, we had no a priori hypotheses or intentions regarding the level of compliance with the app. Rather, we sought to explore and understand how participants used the app and how they used it. Platform use and engagement data collected using secure software analytics while participants use the app will be guided by the AMUsED framework [22]. All interactions with the app are date and time stamped and collected via Matomo click analytics software [25] to help us understand how a participant uses the app; it is open source and is General Data Protection Regulation compliant. All the analytical data were stored securely. The data were stored on a secure server hosted by the University of Manchester. Analytics can be viewed by the researcher using a secure web interface dashboard. Multimedia Appendix 2 provides an overview of the study objectives and time points for the evaluation of each outcome.

### The Intervention: i-Minds App

The i-Minds app was developed in phase 2 of the broader program of our work on TASA. It is a modular intervention, underpinned by mentalization principles, and designed to be used as a standalone platform without restrictions. Following the overall structure and content of a mentalization-based manual developed by members of our team in a previous trial [17], the i-Minds app involves several tasks that not only provide psychoeducation about mentalization but also encourage the application of mentalization principles to a range of scenarios presented to YP. For example, we present the story of a young person with lived experience of TASA (called *Mel*) and, in vignette style, invite users to put themselves in *Mel’s* shoes and consider a range of questions, such as *How do you think Mel feels at school, at home and in other situations that might remind her of what she went through?* We extend the vignette further throughout the app and invite users to pick out situations that could bring about negative feelings for *Mel* in a range of contexts (school, home, and peer relationships), which we further extended using video material and other interactive exercises.

The aim of the intervention is to help YP-TASA understand the motives of adults and peers more clearly, help protect them from future abuse, and help them feel more confident in ambiguous and challenging interpersonal interactions. The app includes content that aims to introduce the concept of mentalization and relate it to scenarios that YP-TASA might identify with, encourage emotional and cognitive literacy in interpersonal interactions, encourage reflection on interpersonal relationship patterns and their development, and explore how these concepts affect emotional expression, behavior, mental health (eg, anxiety, mood, trauma responses, self-esteem, self-harm behavior), and perspective taking. The content is organized into four modules or topic areas: (1) mentalization, (2) psychoeducation of TASA, (3) emotional and mental health, and (4) trauma. There are links to other areas of the app within each core topic area to maximize interactivity and user experience. In addition to the core topic areas, there is a multimedia repository of available resources that can be accessed from the home screen (Multimedia Appendix 3).

The app can either be downloaded onto a participant’s own smartphone or a participant will be loaned a smartphone with the i-Minds app preloaded. The participant can configure certain features of the app themselves during the app “onboarding” session with the researcher, which will either take place in person or remotely, depending on the participant’s choice. Participants were required to work through the mentalization module, which is core to the theoretical orientation of the intervention, before all other features of the app can be accessed. There were no limits to how often or when a participant could use the app. That is, we did not restrict how users interacted with the app over the 6-week intervention period.

Consultation with our advisory group indicated that YP would like the app built around a tree metaphor (see Multimedia Appendix 4 for screenshots of the i-Minds app). Topic areas are represented by branches of a tree, and subtopics are represented by smaller tree branches. As a user works through a subtopic, a leaf is added to the tree, which begins to flourish as the user works through the content (the branches are bare to start with). As suggested by both our young persons’ and professionals’ advisory groups, we also included a “progress ring” around each topic area, which shows the user how far they have progressed through each topic. Tapping on a particular topic area opens an introduction screen that includes introductory text on the topic area. Informed by our advisory group, the flow of each topic area thereafter includes text, video material, and interactive exercises exploring the topic areas, as shown in Multimedia Appendix 3.

### Daily Prompt

A daily prompt in the form of a standard app notification (similar to a Facebook or Twitter notification) will request the participant to check in with the app. The daily notification is designed to prompt engagement with the app and has been included in response to advisory group feedback and our prior experience with app studies [26]. If a participant could not interact with the app at the time of the prompt, they received a reminder notification 3 hours later. This daily prompt was not sent if the participant had already used the app on that day. Participants can also self-initiate use of the app at any time of the day, as many times as they chose. At midday after each 7-day period, an additional weekly prompt invites the user to reflect on app use in the previous week. A research worker will also call the participant 1 week after the onboarding session to troubleshoot the potential technical difficulties. We include the 1-week phone call, as we know that if any technical issues arise, they will most likely be apparent at this time point.

### Onboarding Session

A research worker will meet the participant (either remotely or in person) for a single onboarding session. Here, the research worker will take the participant by configuring the app (eg, including contact details of trusted others), explaining the terms of use in young person friendly terms, demonstrating the features of the app, and demonstrating how to use a smartphone.

### Postintervention

At the end of the 6-week intervention period, if the participant had received a loaned phone, the researcher collected the handset at the follow-up assessment. If the app is downloaded onto a participant’s own handset, the researcher will either delete the app from the participant’s phone so that they can no longer interact with it or the app will automatically stop working no later than 3 weeks after the intervention window. Data network charges will be covered by vouchers and topped up by a member of the research team (if required). Participants will be encouraged to continue receiving support from their health care providers. As participating in this trial does not replace usual care and as all participants will be involved with a clinical service throughout the duration of the trial, participants’ posttrial will continue to be supported by the referring health care provider. Participants will be given an information sheet, signposting them for further support if required.

### Feature Implementation

We followed an agile development process that supports close collaboration between software engineers, clinical academics, and advisory groups that developed and shaped the content of the intervention, enabling the incorporation of changing requirements throughout app development. In line with this process, the working software was delivered on a regular basis and reviewed by the clinical team and advisory groups who provided interim feedback on the user interface developed, software performance, and usability. Any required changes were then incorporated into the next iteration of platform development and continued until the app was fully functional.

i-Minds was written as a hybrid app (HTML, Cascading Style Sheets, JavaScript) using the ionic or capacitor platform [27]. Phone interactions, such as notifications and alarms, are implemented using the capacitor plug-in framework, which facilitates interactions between JavaScript code and platform-specific phone capabilities. The app uses a secure connection (http secure or SSL) to send data to a server. The only data sent were to indicate progress through each module. Before release, the app is unit, integration and system tested, and then reviewed internally at the University of Manchester before submission to the Google Play and Apple App stores.

The i-Minds app is made available on Apple iPhone Operating System and Android devices and implements best practice guidelines offered by the platform providers (Android App Accessibility Guidelines [28] and Apple Human Interface Guidelines [29]). The app is offered as an app to both Google Play and Apple App stores. This software was developed by the University of Manchester DHS.

The app is not a medical device as it has not been designed to be used for diagnosis, prevention, monitoring, treatment, or alleviation of disease, injury, or handicap.

### Accessibility Statement

Accessibility needs of the target audience have been considered when developing accessibility provisions within the i-Minds app, and every effort has been made to ensure that the provision meets the needs of these users. Accessibility needs have been developed in accordance with Public Sector Bodies (web sites and mobile apps) (No 2) Accessibility Regulations 2018 (“Accessibility Regulations” [30]). When using the app on Apple iPhone Operating System and Android devices, users can use the built-in accessibility settings to adjust font sizes, use high contrast settings, zoom in up to 300% with the text staying visible on screen, navigate using only a keyboard or speech recognition software, and use the screen reader. In line with user input, we have made the text in the app as simple as possible to understand to increase its suitability for learners with a wide range of educational backgrounds. Users and research workers can report problems directly to the University of Manchester DHS team email address, which is monitored during the usual working hours. All participants had to accept the end-user license agreement, which is based on a standard template, before they could use the app. A lay version of the end-user license agreement was developed by the research team and was shared and discussed during the onboarding session to ensure that participants were making an informed choice about how their data were used.

### Testing

We followed a strict user-acceptance testing procedure before the first public test release of the app. This procedure includes a section that specifically encourages researchers to test all accessibility features of their devices and determine which aspects of the app support these features. This procedure highlights any content that is not compliant with the Web Content Accessibility Guidelines 2.1-AA standard. Any noncompliant content that is considered the core functionality of the app or essential to the user experience was addressed at this stage.

### Privacy, Security, and Risk

Security measures are robust and fully comply with national policies and relevant data management and information governance policies and procedures of the participating Universities and NHS organizations. All trial investigators and site staff comply with the requirements of the Data Protection Act 2018 or General Data Protection Regulation and the NHS Confidentiality Code with regards to the collection, storage, processing, and disclosure of personal information and will uphold these acts’ core principles. The i-Minds app uses the University of Manchester Privacy Notice for Research Participants (available upon request). Once the user has installed it, they are given a unique code. The app will only become functional after the unique code has been given to the researcher, and the researcher will add it to the participant record via the web interface.

The following security features are used: (1) app: Use of the app requires a PIN code on the user’s phone, which will be checked and enforced when the app starts. Data collected within the app are sent to the server over http secure using secure ciphers; (2) server: the server is part of the University of Manchester’s research virtual machine infrastructure and is secured inside the University’s network. Operating system and related security patches are applied on a regular basis to these servers by the University’s infrastructure team; and (3) web-based interface: members of the research team are each allocated their own username and password to access the web-based interface. The DHS team has administrative access to the web-based interface to enable it to maintain the system and manage accounts for researchers. All members of this team received their current data protection training.

To minimize security risks, no part of the system will collect identifiable information. A pseudonymized identifier was used to identify each participant. Only the research team knows the link between this identifier and the actual participant. This link will not be stored anywhere in the system, and the software team will only have access to pseudonymized identifiers.

Regarding participant safety and risk, at the point of referral, we will collect the contact details of the participant’s general practitioner and key worker from the referring service, who will be our primary point of contact for subsequent liaison with the clinical team or key worker to manage any significant risk. If a participant discloses something that raises serious concerns (eg, historical abuse, current abuse) about their safety or the safety of others or if a research worker becomes distressed themselves, given the topic and nature of the work, we will follow the safeguarding and distress management procedures described in our coproduced and ethically approved detailed standard operating procedure. As a general rule, we signed participants to the appropriate sources of support in their locality.

### Withdrawal

A participant will be withdrawn if they express either to a member of the research team or to their key clinical worker that they wish to withdraw from the intervention, the trial, or both. If a participant withdraws from the study, we will complete a withdrawal form, suspend data collection activities, and retain and analyze the anonymized data collected up to the point of withdrawal. If a participant withdraws from the intervention, we will cease their use of the app but still invite them to complete follow-up measures and a qualitative interview to understand their reasons for withdrawal from the intervention (but only if the participant consents to this).

### Independent Oversight

Independent oversight of the trial is provided by a project steering committee (PSC) and a DMEC who are independent from the sponsors and investigators and approved by the funder. The PSC comprises an independent chair, statistician, academic, clinician, and patient and public involvement representative and will meet every 6 months over the duration of the project. The PSC is responsible for the independent oversight of the project on behalf of the sponsor and funder and will ensure that the project is conducted according to the rigorous standards set out in the Department of Health’s Research Governance Framework for Health and Social Care and the Guidelines for Good Clinical Practice. The DMEC comprises an independent chair, statistician, and digital health expert and is responsible for monitoring ethical issues of consent and confidentiality, data quality and completeness, incidence of adverse events, compliance with the protocol by participants and investigators, and any other issues relevant to the transparent and ethical delivery of the trial. The DMEC aims to meet on 3 occasions over the course of a feasibility clinical trial.

### Patient and Public Involvement and Engagement

Our aim was to develop a digital intervention as opposed to an “offline” one that was directly influenced by the views and preferences of YP-TASA. Before our application for funding, we conducted extensive consultations with YP-TASA and a range of stakeholders. In line with NICE [1] research recommendations for developing interventions to improve well-being following TASA, we have continued to seek extensive feedback from YP-TASA, parents, and practitioners via 3 advisory groups, who we will work with for the lifetime of feasibility and nested qualitative studies. The active involvement of YP-TASA, their parents or caregivers, and relevant professionals ensures active input into the development of the intervention, including its content, format, and structure, and linked procedures to ensure participant safety, as well as to deliver its evaluation and dissemination of the findings to maximize impact.

Three advisory groups were established. A national stakeholders advisory committee meets every 6 months and comprises professionals from national law enforcement and child protection or safeguarding organizations, academic institutions, TASA support services, and social media organizations. A professional advisory committee meets every 6 months and comprises clinicians from relevant services that provide mental health and web-based sexual abuse or safeguarding support to YP in our recruiting sites. Our lived experience advisory group comprises up to 10 people with TASA experiences who meet monthly to bimonthly over the course of the project. Input was received both through web-based meetings and through individual written feedback on participant-facing documents and app content itself. The lived experience advisory group has already influenced the participant information sheet, for example, explaining that not all sexual experiences on the web are unwanted and some may be wanted but in fact go wrong; as such, we have reflected this important point in our participant-facing documents. Our advisory groups reviewed and entered key trial documents, including our risk management and distress protocols.

In line with the United Kingdom Government User Research guidelines [31], app development was supported by a series of participatory consultations, which enabled end users to influence the design and functionality of the app, leading to the development of an intervention that we hope is more likely to be acceptable, have better uptake, and be effective. By working together, developers and users can learn and optimize platform functionality, with developers being responsible for pointing out technical options and users providing information about their needs, practices, and how they will use the system.

### Statistical Analysis Plan

Because hypothesis testing is not the objective of this study, formal power calculations are not appropriate. The proposed sample size is sufficient to establish feasibility and to report response rates, follow-up rates, safety information, and attrition, as well as the clinical characteristics of our study population at the beginning of the study and follow-up.

Appropriate descriptive statistics (mean and SDs for continuous data; counts and percentages for categorical data) will be used to summarize (1) recruitment and retention data in line with CONSORT statement standards and the extent to which services refer to trial, (2) data use patterns (frequencies; data visualization) using secure software analytics in line with the AMUsED framework for analyzing and measuring use and engagement data in digital interventions [22], (3) completeness of study measures, and (4) the number and nature of adverse events observed over the course of the trial. Pre- and postintervention questionnaire data will be analyzed using the Leeds Reliable Change Indicator [32] or other approaches for the evaluation of reliable or clinically significant changes in secondary outcome measures (eg, when conditions for the application of the Leeds Reliable Change Index are not met) to determine the number and proportion of participants who achieved significant improvement on clinical measures and who achieved no significant worsening of clinical measures at posttreatment. To explore how different participants engage with the app, attrition, and clinical benefit across key demographic groups (eg, LGBTQ+ and BAME) and potential service differences across recruitment sites, we will use descriptive analysis to inform future work; we will not test for differences, as the study is not powered for this.

When a participant has completed a questionnaire but has not completed all elements of that questionnaire, we will use validated instruments and will follow the established procedures for calculating overall scores in the presence of partially missing data.

When data are completely missing for a whole questionnaire, we will record the occurrence of this and, if the prevalence of such missing data permits, use multiple imputations assuming these data are missing at random. We may also, if we think these data may be informatively missing, ie, missing not at random, investigate appropriate sensitivity type analyses to see whether our findings are robust to these missing data.

For qualitative data, we used a qualitative framework approach. Analysis of qualitative interviews will occur alongside transcription and data collection so that we can iterate our topic guide. We initially use the framework method to adopt an inductive approach to theme generation. Subsequent theme refinement will be deductive and guided by the NPT. NPT is a widely used [33,34] theory to explain the processes by which an intervention becomes, or fails to become, normalized into routine practice, and offers a framework for assessing the conditions in which interventions become practically workable in health care. NPT comprises 4 constructs (coherence, cognitive participation, collective action, and reflexive monitoring), which are a set of propositions that we will use to explore perceptions, expectations, attitudes, challenges, and unintended consequences of integrating a digital intervention for YP-TASA in existing NHS service and e-therapy provider pathways. This approach enables us to answer our research questions while allowing important insights to be iteratively produced. The coding framework is then applied to the analysis of subsequent transcripts, with ongoing adaptations as new themes emerge. Data will then be charted into a matrix with illustrative extracts and interpretive themes refined through discussions at regular analysis meetings. We will engage a wider research team, our advisory groups, and stakeholders in the analysis process (member checking or participant verification). This peer verification process (together with member checking) is a recognized method for ensuring the trustworthiness of the data and subsequent findings [35]. Records of field notes will be maintained and reflections providing adjunctive data will be used to illuminate and justify interpretative decisions.

### Dissemination

We plan to present our findings at academic conferences and publish them in peer-reviewed academic journals. We also developed a project website [36] which we will keep up to date with study progress. Our social media account will also be used to promote the study, update progress, and disseminate high-level findings. The findings will also be disseminated with support from our advisory groups in a multimedia format at, for example, practitioner and public forums, events (eg, conferences, meetings, seminars), and via government and policy events. Feedback to participants and clinical services will be provided through coproduced written summaries.

## Results

We aimed to collect data from up to 60 YP. This study was registered as a clinical trial (ISRCTN43130832). Recruitment was initiated in May 2022. We expect to report results for feasibility on (but not necessarily limited to) (1) the number of eligible participants consenting; (2) the total number recruited, including information about recruitment setting to inform sampling strategy of a future definitive trial; (3) completeness of outcome measures; (4) the number lost to follow-up; (5) the number of services offered the intervention; (6) the proportion of participants completing the intervention; (7) dropout rates and reason for withdrawal; (8) platform use and engagement data; and (9) safety of the app and our trial procedures. We also expect to report on differences in engagement and attrition and potential clinical benefits across key demographic groups (eg, LGBTQ+ and BAME), the demographics and service differences across recruitment sites, and the barriers and enablers to integration and uptake into existing mental health care provider pathways. We expect the results to be available at the end of 2023 (subject to recruitment targets and funding requirements).

## Discussion

### Expected Findings

The aim of our NIHR-funded i-Minds trial was to assess the feasibility, acceptability, safety, and usability of the theory-driven, coproduced, mentalization-based i-Minds app for YP-TASA. Specifically, our primary objectives were to determine (1) whether it is feasible to deliver the i-Minds app for YP-TASA, including recruitment and retention to the trial and identifying primary and secondary outcome measures; (2) whether the i-Minds app is acceptable to users; (3) whether the i-Minds app is usable and safe; and (4) how best to integrate the app into the existing NHS and web-based mental health and sexual abuse service care pathways. To the best of our knowledge, this is the first clinical trial of this type in this population worldwide, which is why we chose to conduct a feasibility clinical trial rather than a powered efficacy trial.

To date, we have carried out a series of qualitative interviews and focus groups with HCPs working with YP in secondary care mental health settings, held regular advisory group meetings with our 3 advisory groups, incorporated input and feedback from this work into the development of the app content and user interface, built and tested the app and met all the security standards required for deployment, and commenced recruitment to the clinical feasibility trial. The user-driven design approach we have adopted in this trial is intended to ensure that the intervention is engaging and relevant and reflects the experiences of YP-TASA. For example, Vichta et al [37] used interactive workshops and a web-based survey to gather YP’s perspectives on platform integration into mental health care. Venning et al [38] conducted semistructured interviews and focus groups to explore the acceptability of a low-intensity digital computer-based training platform incorporating a virtual coach with university students and HCPs.

If feasibility for the current approach in this population is found, we will use the results from the nested qualitative study about how our approach can be integrated into a model of care (eg, hybrid, stepped, or blended) to inform the design of the next phase trial, should one be indicated.

### Limitations

This study targeted young help-seeking individuals accessing clinical services. It is hoped that these findings could also be useful in YP experiencing distress but not necessarily seeking help or support from clinical services. As this was a pilot study, we were unable to conclude whether the intervention brought about a clinically significant change. However, this study design, with its focus on feasibility, acceptability, safety, and usability, will provide valuable insights that will inform parameters for a future definitive trial, should this be indicated. Moreover, feedback from all stakeholders will allow for improvements in the app, trial procedures, and plans for implementation.

### Conclusions

This feasibility clinical trial stands to advance the support for YP-TASA, a nascent area for both research and clinical delivery. Exploring the feasibility, acceptability, usability, and safety of the intervention is the first step in creating a more definitive intervention that may be further tested in future studies and implemented to support YP-TASA across a range of settings.

## Appendix

Multimedia Appendix 1 Schedule of quantitative assessments.

Multimedia Appendix 2 Overview of the study objectives and time points of evaluation of each outcome.

Multimedia Appendix 3 Summary of topic areas and app features.

Multimedia Appendix 4 Screenshots of the i-Minds app.

## Abbreviations

BAME: Black, Asian, and minority ethnic
CAMHS: Child and Adolescent Mental Health Services
CONSORT: Consolidated Standards of Reporting Trials
CSA: child sexual abuse
DHS: Digital Health Software
DMEC: Data Monitoring and Ethics Committee
HCP: health care professional
HRA: Health Research Authority
LGBTQ+: lesbian, gay, bisexual, transgender, queer, and others
MBT: mentalization-based therapy
NHS: National Health Service
NICE: National Institute for Clinical Excellence
NIHR: National Institute for Health and Care Research
NPT: Normalization Process Theory
PSC: project steering committee
REC: Research Ethics Committee
REDCap: Research Electronic Data Capture
TASA: technology-assisted sexual abuse
YP: young people
YP-TASA: young people who have experienced technology-assisted sexual abuse

## Data Availability

The data set generated in this study is not publicly available owing to ethical restrictions for personal health information; however, limited, deidentified data may be made available from the corresponding author on reasonable request.
