# Supplementary material for: A Digital Intervention to Improve Mental Health and Interpersonal Resilience in Young People Who Have Experienced Technology-Assisted Sexual Abuse: Protocol for a Nonrandomized Feasibility Clinical Trial and Nested Qualitative Study
Source: JMIR Res Protoc. 2023 Mar 21;12:e40539. doi: 10.2196/40539 (PMC10131936; doi:10.2196/40539)
Supplement: Multimedia Appendix 1 [file resprot_v12i1e40539_app1.docx]

**Multimedia Appendix 1.** Schedule of Quantitative Assessments.

| **Assessment** | **Concept measured** | **Baseline** | **Follow-up** |
| --- | --- | --- | --- |
| Sociodemographic Questions* | - | X |  |
| Clinical Information* | - | X |  |
| Technology Use, Experience of Online harms, Help-seeking |  | X |  |
| RFQ-Y | Mentalisation | X | X |
| PRIUSS | Problematic internet use | X |  |
| RCADS-25 | Emotional distress | X | X |
| CRIES* | Online-abuse related distress (by anchoring the CRIES, to the young person’s OSA experience) | X | X |
| DERS | Emotion regulation | X | X |
| ISM | Interpersonal Sensitivity | X | X |
| ECR-RC | Views/attitudes towards close interpersonal relationships | X |  |
| Connor-Davidson Resilience Scale (CD-RISC-10) | Resilience | X | X |
| App satisfaction questionnaire | App satisfaction / acceptability |  | X |
| Matomo click analytics | Usage monitoring | Throughout the trial | |

Reflective Functioning Questionnaire for Youths (RFQ-Y); Problematic and Risky Internet Use Screening Scale (PRIUSS); Revised Child Anxiety and Depression Scale–25 item version (RCADS-25); Child Revised Impact of Events Scale (CRIES); Difficulties in Emotion Regulation Scale-Short Form (DERS-SF); Interpersonal Sensitivity Measure (ISM); Experiences in Close Relationships Scale–Revised Child version (ECR-RC); Connor-Davidson Resilience Scale-10 item version (CD-RISC-10); Matomo Click Analytics (<https://matomo.org/>).

*Demographic, clinical, and CRIES are administered with the support of the research worker. All other measures can be administered either with support of the research worker or self-directed.
