# Supplementary material for: A Digital Intervention to Improve Mental Health and Interpersonal Resilience in Young People Who Have Experienced Technology-Assisted Sexual Abuse: Protocol for a Nonrandomized Feasibility Clinical Trial and Nested Qualitative Study
Source: JMIR Res Protoc. 2023 Mar 21;12:e40539. doi: 10.2196/40539 (PMC10131936; doi:10.2196/40539)
Supplement: Multimedia Appendix 2 [file resprot_v12i1e40539_app2.docx]

**Multimedia Appendix 2.** Overview of the study objectives and timepoints of evaluation of each outcome.

| **Objectives** | **Outcome Measures** | **Timepoint(s) of evaluation** |
| --- | --- | --- |
| **Primary Objective** To test the feasibility of delivering the digital intervention (app) for YP-OSA including the extent to which services refer to the study  To test the acceptability of the app | Recruitment and retention data congruent with all relevant fields of the CONSORT statement for feasibility studies  Acceptability – in-depth interviews to examine whether expectations met, level of support needed to engage with the app, overall impressions, likes / dislikes about the app, how it helped / did not help, perceived changes, barriers to participation / engagement | Throughout the trial |
| **Secondary objectives**  To explore whether the app brings about clinically meaningful change in outcomes  To explore differences in engagement and potential clinical benefit across key demographic groups  To explore barriers and enablers to integration and uptake into existing NHS clinical services  To test the usability and safety of the app | Battery of questionnaires measuring mentalisation, problematic internet use*, emotional distress, online abuse-related stress, emotion regulation, interpersonal sensitivity, views/attitudes towards close interpersonal relationships* and resilience.  Registration form requesting demographic (e.g. gender, ethnicity, age, sexual orientation, internet use, level of social deprivation) and clinical (e.g. diagnosis, treatment regime in referring service, other sources of support) details.  Qualitative interviews with HCPs and service managers from referring services to examine ways to maximise uptake, utility, user experience, acceptability, satisfaction, reach of the app; how the app can be locally adapted and translated into practice; referral routes to the app via existing care pathways; strategic perceptions about whether the app can be scaled up.  Usability – proportion of participants completed intervention, dropout rates, reason for withdrawal, app usage and engagement (using secure software analytics guided by AMUsED framework).  Safety - detailed adverse events reporting procedure. | Baseline and 7-9 weeks post-baseline  (*7 weeks only) |
