# Supplementary material for: A Digital Intervention to Improve Mental Health and Interpersonal Resilience in Young People Who Have Experienced Technology-Assisted Sexual Abuse: Protocol for a Nonrandomized Feasibility Clinical Trial and Nested Qualitative Study
Source: JMIR Res Protoc. 2023 Mar 21;12:e40539. doi: 10.2196/40539 (PMC10131936; doi:10.2196/40539)
Supplement: Multimedia Appendix 3 [file resprot_v12i1e40539_app3.docx]

**Multimedia Appendix 3.** Summary of topic areas and app features.
